# Mitigation of Tacrolimus-Associated Nephrotoxicity by PLGA Nanoparticulate Delivery Following Multiple Dosing to Mice while Maintaining its Immunosuppressive Activity

**DOI:** 10.1038/s41598-020-63767-1

**Published:** 2020-04-21

**Authors:** Aws Alshamsan, Ziyad Binkhathlan, Mohd Abul Kalam, Wajhul Qamar, Hala Kfouri, Mohammed Alghonaim, Afsaneh Lavasanifar

**Affiliations:** 1 Nanobiotechnology Unit, College of Pharmacy, King Saud University, P.O. Box 2457, Riyadh, 11451 Saudi Arabia; 2 Department of Pharmaceutics, College of Pharmacy, King Saud University, P.O. Box 2457, Riyadh, 11451 Saudi Arabia; 3 Faculty of Pharmacy and Pharmaceutical Sciences, University of Alberta, Edmonton, Alberta T6G 2H7 Canada; 4 Central Laboratory, College of Pharmacy, King Saud University, Riyadh, Saudi Arabia; 5 Department of Pharmacology and Toxicology, College of Pharmacy, King Saud University, Riyadh, Saudi Arabia; 6 Department of Pathology, College of Medicine, King Saud University, Riyadh, 11451 Saudi Arabia; 7 King Salman Bin Abdulaziz Chair for Kidney Disease, King Saud University, Riyadh, 11451 Saudi Arabia; 8 Department of Chemical and Material Engineering, University of Alberta, Edmonton, Alberta T6G 2V4 Canada

**Keywords:** Immunology, Drug delivery, Medical toxicology

## Abstract

The aim of this study was to assess the ability of PLGA nanoparticles (NPs) to reduce the tacrolimus (TAC)-associated nephrotoxicity following multiple dose administration. The mean diameter of prepared NPs was in the range of 227 to 263 nm with an 8.32% drug loading (w/w). Moreover, *in vitro* release profile of TAC-loaded NPs showed a sustained release of the drug with only less than 30% release within 12 days. Flow cytometry as well as fluorescence microscopy results confirmed the uptake of FITC-labelled PLGA NPs by dendritic cells. The *ex vivo* study showed that TAC-loaded NPs caused a significant suppression of the proliferation of CD4^+^ and CD8^+^ cells, which was comparable to the control formulation (Prograf). *In vivo* immunosuppressive activity as well as the kidney function were assessed following drug administration to mice. The animals received TAC subcutaneously at a daily dose of 1 mg/kg for 30 days delivered as the control formulation (Prograf) or TAC-loaded NPs. The results revealed significantly lower drug-associated toxicity with an activity comparable to Prograf for TAC-loaded PLGA NPs. These findings show a potential for PLGA NPs in reducing the nephrotoxicity of TAC while preserving the immunosuppressive activity.

## Introduction

Tacrolimus is a potent immunosuppressive agent used clinically to reduce the risk of graft rejection in post-operative transplants patients^1–3^. It acts by suppressing interleukin-2 (IL-2) production in T-cells^4^, and also by inhibiting phosphatase activity of calcineurin through binding to the intracellular immunophilin FKBP-12^5^. The currently available intravenous formulation of TAC (Prograf) contains HCO-60 (PEGylated castor oil) as a surfactant, which is reported to cause several side effects including hypersensitivity reactions^6–8^. TAC is available in oral dosage forms including immediate release capsules (Prograf), extended release capsules (Astagraf XL and Advagraf), and extended release tablets (Envarsus XR). Several adverse effects have been reported with the use of TAC including hypertension, pruritus, leucocytosis, neurotoxicity, nephrotoxicity, cardiotoxicity and hepatotoxicity^9–13^.

Drug-induced nephrotoxicity is the major dose-limiting side effect of TAC with a reported overall incidence as high as 44%^14^. Unfortunately, nephrotoxicity can lead to severe complications such as negative impact on graft survival and life expectancy of the patients. Indeed, nephrotoxic effects present challenges during therapeutic regimen with these drugs^15,16^. Several strategies have been proposed to address this problem. Dose reduction is one of the strategies that has been evaluated by clinicians along with using other agents such as sirolomus or everolimus, which are found to be effective in improving long-term effects in renal transplant cases^17,18^.

Several formulations have been investigated to improve the aqueous solubility of TAC, enhance its efficacy, and reduce its toxicity^19–23^. Polymeric nanoparticles (NPs) are a promising effective alternative to traditional drug formulations. Poly(lactic-*co*-glycolic acid) (PLGA) is a family of biodegradable copolymers that has been approved by the US FDA and European Medicine Agency (EMA) for biomedical applications^24,25^. This polyester undergoes hydrolysis and the ultimate degradation products are lactic acid and glycolic acid, which are endogenous compounds and easily metabolized by the body via the Krebs cycle^24^. The biocompatibility and safety profile of PLGA copolymers are well documented, and make them suitable carriers for drug delivery applications^26–28^. Indeed, PLGA NPs have been successfully used for encapsulating various low and high molecular weight drugs and biologics^29–32^. Encapsulation of drugs or macromolecules (e.g. peptides and proteins) into PLGA NPs have been shown to enhance the stability, control the release, and prolong the circulation time of the payload in the blood. Furthermore, PLGA NPs have also been shown to deliver antigens to dendritic cells^33^. So far, the extensive research on the application of PLGA as drug delivery system have resulted in several marketed formulations (injectable implants) for human use^34,35^.

We have previously shown that PLGA NPs are capable of loading different types of payloads including small molecular weight drugs^36–38^, large molecular weight molecules^39,40^, as well as siRNA^41^. The drug-loaded PLGA NPs always exhibited diameters in the nano range, high loading efficiency, and sustained release of the payload *in vitro*. Moreover, we have recently shown that TAC-loaded PLGA NPs were able to enhance the *in vivo* ocular bioavailability and nearly doubled the elimination half-life of the drug in aqueous humour compared to TAC aqueous suspension^38^.

The main objective of the current study was to assess the potential of PLGA NPs formulation of TAC in enhancing the therapeutic index of TAC in rodents mainly by reducing the drug’s nephrotoxicity. Nephrotoxicity in mice was investigated by assessing the renal function parameters in serum and by histopathological examination of kidney tissues following multiple dosing of TAC-loaded PLGA NPs in comparison to a control formulation (Prograf). The *in vivo* immunosuppressive activity of TAC-loaded NPs was also assessed using T Cell Proliferation Assay, and the profile was compared to Prograf.

## Results

### PLGA NP characterization

**Figure 1:**
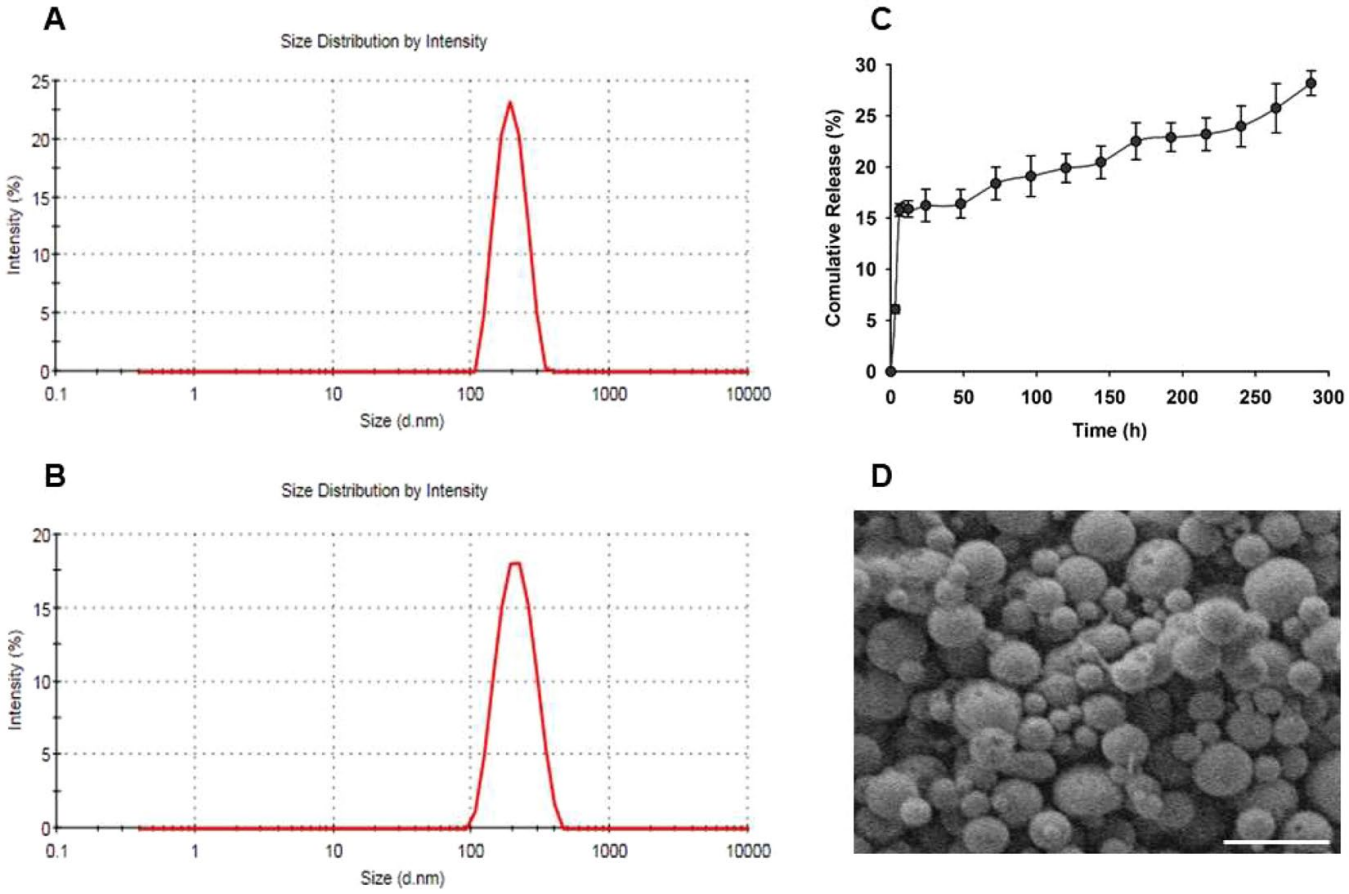
Particle size distribution analysis of PLGA-NPs by DLS for empty (**A**) and TAC-loaded (**B**); *In vitro* release profile of TAC-loaded PLGA-NPs in PBS at pH 7.4 (**C**); SEM image of TAC-loaded PLGA-NPs (**D**) [Scale bar represents 500 nm].

The mean diameter of empty PLGA NPs and TAC-loaded NPs were 227.4 ± 10.4 nm and 263.3 ± 14.8 nm, respectively (Fig. 1A,B, respectively). This particle size range is suitable for the intended delivery of TAC (i.e. macrophages and dendritic cells uptake). Polydispersity indices, which measure the width of particle size distribution, were 0.254 ± 0.034 and 0.114 ± 0.008 for empty NPs and TAC-PLGA NPs, respectively. These values suggest that the prepared NPs were unimodal with a relatively narrow distribution (Fig. 1A,B). The zeta potential of empty PLGA NPs and TAC-PLGA NPs were −3.16 ± 0.83 mV and −10.53 ± 1.42 mV, respectively. The encapsulation efficiency and drug loading of TAC in the optimized PLGA-NPs was found to be reasonably high (84.6% and 8.3%, respectively). High amount of TAC incorporation and loading might be attributed to the rapid quenching of TAC into the matrix of PLGA due to the good stabilizing property of PVA and Poloxamer-188. The *in vitro* release of TAC from TAC-loaded PLGA NPs was studied in PBS (pH 7.4). The profile showed a burst drug release within the first few hours (i.e. up to 6 h), which was followed by a slow and sustained release of the drug for 288 hours (12 days) which did not exceed 30% of the incorporated drug at this time point (Fig. 1C). SEM images of the NPs revealed that the obtained PLGA NPs have solid dense spherical structures with smooth surfaces (Fig. 1D).

### *In vivo* immunosuppression in mice

**Figure 2:**
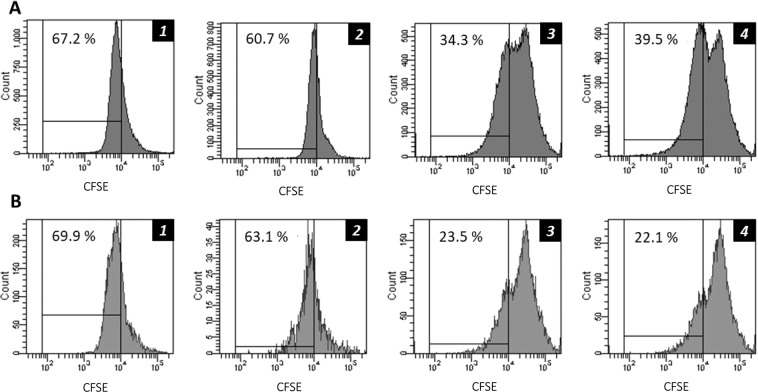
*In vivo* suppression of T cell proliferation in male mice following a daily subcutaneous injection (for 7 days) of either (1) Normal Saline, (2) Empty PLGA NPs, (3) Prograf, or (4) TAC-loaded PLGA NPs. Flow cytometry analysis was conducted on two cell groups: (**A**) CD4^+^ T cells and (**B**) CD8^+^ T cells (n = 6). The percentage of proliferating T cells is presented next to each gate. All samples are composed of the same number of acquired events (10^6^ cells).

Administration of Prograf or TAC-loaded PLGA NPs caused significant immunosuppression reflected in the reduced number of CD4^+^ and CD8^+^ cells in mice analysed by flow cytometry. Figure 2A presents effects on CD4 + cells in mice after administration of normal saline, empty PLGA NPs, Prograf, and TAC-loaded PLGA NPs. Prograf and TAC-loaded PLGA NPs caused CD4^+^ cell suppression by 48.95% and 41.22%, respectively (Fig. 2A3,A4). The effects of these treatment groups on CD8^+^ cells are shown in Fig. 2B. The % suppression of CD8^+^ cells were found to be 66.4% and 68.4% for Prograf and TAC-loaded PLGA NPs, respectively (Fig. 2B3,B4). Empty PLGA NPs also exhibited slight suppression of CD4^+^ (9.67%) (Fig. 2A2) and CD8^+^ (9.72%) cells (Fig. 2B2) when compared with normal saline group mice (Fig. 2A1,B1).

### *In vivo* TAC nephrotoxicity evaluation in rodents

#### Histopathological evaluation

**Figure 3:**
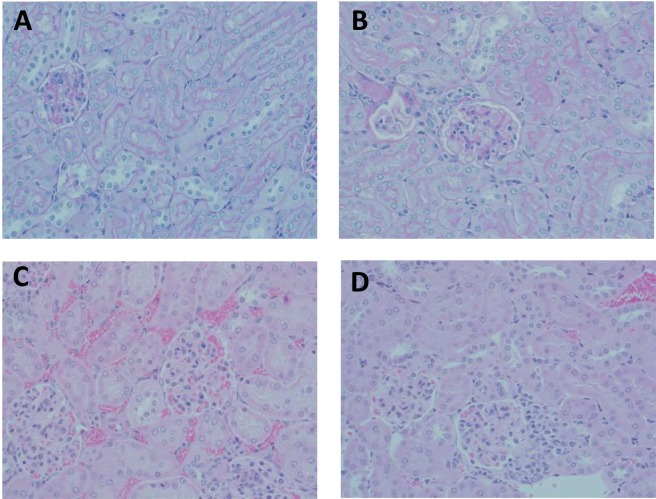
Histopathology of mice kidney from different treatment groups (n = 6): (**A**) Normal Saline group: shows normal histology; (**B**) Empty PLGA NPs group: mild distension of the renal glomerulus capsular space and minimal interstitial oedema; (**C**) Prograf group: high glomerular congestion and oedema along with renal tubular infiltration (**D**) TAC-loaded PLGA NPs group: glomerular congestion and oedema along with renal tubular infiltration.

Histological observations under microscope reveal minimal interstitial infiltration in mice administered normal saline (Fig. 3A). Administration of empty PLGA NPs appear to exhibit mild distension of the renal glomerulus capsular space and minimal interstitial oedema (Fig. 3B). Prograf administration at the dose of 1 mg/kg caused high glomerular congestion with interstitial infiltration and oedema (Fig. 3C). However, administration of tacrolimus-loaded PLGA NPs did not cause effects similar to that of Prograf. Tacrolimus-loaded PLGA NPs administration had effects lower than that of Prograf but higher than that of normal saline or empty PLGA NPs, exhibiting effects including glomerular congestion and oedema along with renal tubular infiltration (Fig. 3D). Histopathological evaluations were done by a pathologist blinded about the animal groups based on different treatments.

### Assessment of renal function

**Figure 4:**
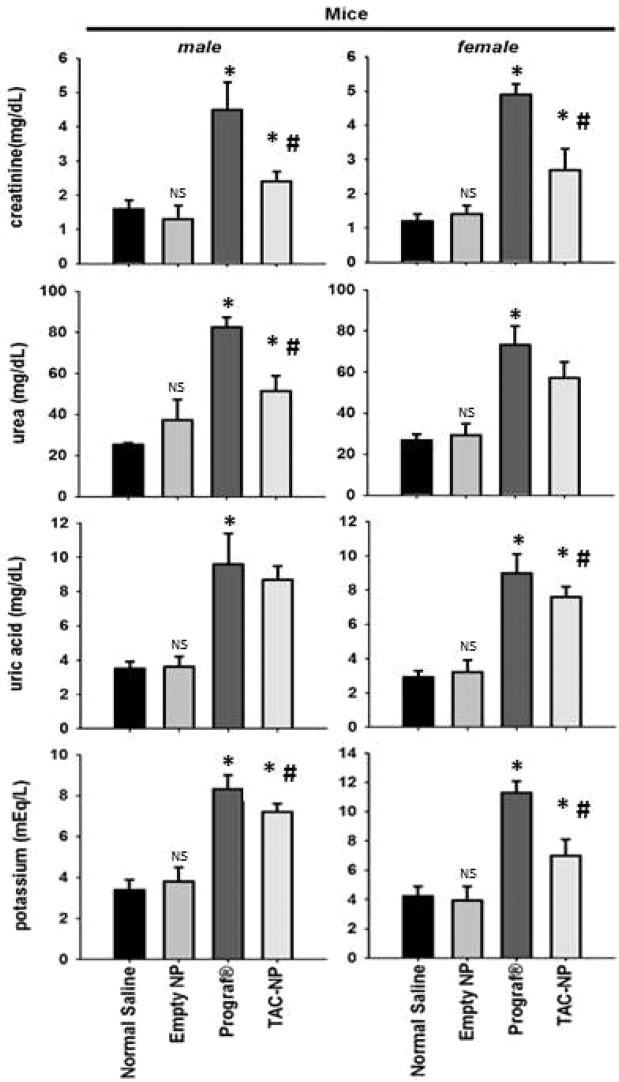
Blood biochemical parameters in mouse serum after 30 days of a daily subcutaneous injection (n = 6). The daily dose of TAC was 1 mg/kg administered as Prograf or TAC-loaded PLGA NPs. *p < 0.05 when compared with Normal Saline group. ^#^*p* < 0.05 when compared with Prograf group. ^NS^Not Significant when compared with Normal Saline group.

Figure 4 shows the serum levels of various biochemical parameters used to assess renal function in mice from different treatment groups. Following a daily subcutaneous injection (1 mg/kg) for 30 days, compared to normal saline group, Prograf group showed a significantly higher values (*p* < 0.05; ANOVA with Dunnett’s *post hoc* test) for all the biochemical parameters (Fig. 4). Although TAC-loaded PLGA NPs group also showed a significantly higher levels for all the parameters compared to normal saline group (*p* < 0.05; ANOVA with Dunnett’s *post hoc* test), the values were significantly lower than those obtained with Prograf (*p* < 0.05). For instance, whereas the baseline mean values of serum creatinine and urea measured in normal saline group (male mice) were approximately 1.5 and 25 mg/dL, respectively, the corresponding values were 4.5 and 85 mg/dL in Prograf group, and 2.3 and 50 mg/dL in TAC-loaded PLGA NPs group, respectively. All the biochemical parameters measured for the empty PLGA group were comparable to those obtained with normal saline group. Moreover, the effects of different treatment groups on the renal function parameters do not seem to be influenced by gender because the profile observed in male mice was similar to that obtained from their female counterpart.

**Figure 5:**
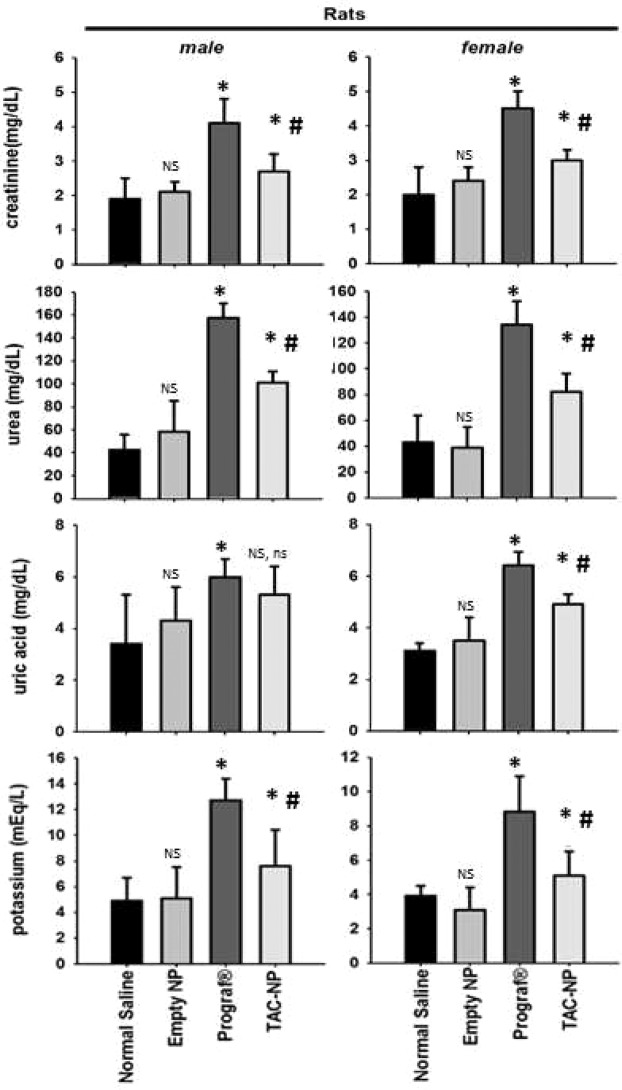
Blood biochemical parameters in rat serum after 30 days of a daily subcutaneous injection (n = 6). The daily dose of TAC was 1 mg/kg administered as Prograf or TAC-loaded PLGA NPs. *p < 0.05 when compared with Normal Saline group. ^#^*p* < 0.05 when compared with Prograf group. ^NS^Not Significant when compared with Normal Saline group. ^ns^Not Significant when compared with Prograf group.

To confirm whether these finding can be achieved in another species, the same study was conducted using male and female Wistar rats (200–250 g). The results were comparable to those obtained in mice. Specifically, TAC-loaded PLGA NPs had lower nephrotoxicity compared to Prograf, and that the effects of different treatment groups on the renal function parameters were comparable between male and female rats (Fig. 5).

## Discussion

TAC is still considered a mainstay in immunosuppressive therapy especially in organ transplantation. It reduces the chances of graft rejection efficiently but has a narrow therapeutic window. Moreover, owing to the non-selective distribution of TAC, several organs have been reported to be affected by the drug’s adverse effects. Specifically, kidneys appear to be primary target organ for TAC-induced toxicities^42^. Moreover, TAC is a low solubility and high permeability drug (BCS class II). Poor water solubility (4‒12 µg/mL), low oral bioavailability, and high intra- and inter-subject variability are among the issues reported for TAC^43^. Low oral bioavailability of TAC is attributed to various factors including low solubility, extensive first-pass metabolism in liver and intestine, and P-glycoprotein-mediated drug efflux^43,44^.

We have recently shown that PLGA NPs were able to enhance the ocular bioavailability and significantly prolonged the elimination half-life of the encapsulated TAC in aqueous humour compared to the drug in aqueous solution^38^. The main objective of the current study was to develop a new TAC nanoformulation with PLGA NPs to control the release of the drug and reduce its nephrotoxic effects. The absolute high positive or negative zeta potentials are considered good for the stability of PLGA-NPs. The zeta potential of empty PLGA NPs and TAC-PLGA NPs were −3.16 ± 0.83 mV and −10.53 ± 1.42 mV, respectively, which cannot be considered to create a sufficient electrostatic repulsion among the NPs to prevent their aggregation. Rather, it is the steric stabilization mechanism caused by the external polyoxyethylene (PEO)-fragments (present in Poloxamer-188 which was used as stabilizer in the PLGA-NPs) overhang into the suspension^45^. Therefore, it would be noteworthy to mention here, that not only the electrophoretic mobility (zeta-potential) but also the steric hindrance mechanism that provided the uniform heterogeneous distribution and prevented the aggregation of the PLGA-NPs which in turn provided sufficient colloidal stability to the NPs controlled by steric interactions.

The developed PLGA NPs showed a high encapsulation efficiency reaching around 85%, which translates to about 8% drug loading. These values are higher than those reported by Shin *et al*., where the highest values for encapsulation efficiency and drug loading reached were 60 and 6%, respectively^46^. This higher drug loading in our formulation would allow for administration of a lower amount of PLGA polymer per dose. Moreover, in studies by Shin *et al*., the cumulative release of TAC from their PLGA NPs formulation (at pH 7.4) was around 40% at 96 h and 70% at 240 h^46^. In contrast, our developed formulation showed a better control over drug release with only 16% at 48 h and less than 25% at 240 h. This could be attributed to the higher PLGA concentration used in our study, which may help strengthen the drug-polymer interaction and slow down the hydrolytic degradation of the polymer. Other factors such as the differences in the preparation method, release study design and/or assay method can have a significant impact on the *in vitro* release profile.

Data from PLGA NP characterization showed that the prepared NPs were spherical with a mean diameter of 263.3 ± 14.8 nm, which is within the range that has been reported for TAC-loaded PLGA NPs and shown to be successfully used for lymphatic targeting^46^. Indeed, this was evident in our current study where we showed the uptake of the prepared PLGA NPs by dendritic cells (DCs), *in vitro* (Fig. S1). Since the mean particle size was around 263 nm, the PLGA NPs were likely taken up by DCs via macro-pinocytosis as well as phagocytosis^47^. The fluorescence microscopic image showed the presence of FITC-labelled PLGA NPs in the cytoplasm, in the vicinity of the nucleus and inside the lysosomes (Fig. S1B). Moreover, compared to untreated DCs, the flow cytometry analysis showed a significant increase in the fluorescence intensity of DCs incubated with FITC-labelled PLGA NPs (Fig. S1A).

PLGA NPs have been reported to improve therapeutic efficacy and reduce the toxicity of encapsulated immunosuppressive agents^48^. We showed here that the *in vivo* immunosuppressive effects of TAC-loaded NPs in mice was comparable to that obtained with Prograf. This was based on the similarity in the percentages of reduction in CD4^+^ and CD8^+^ T cell proliferation following the administration of TAC-loaded NPs or Prograf to mice (Fig. 4). Interestingly, the empty PLGA NPs showed a slight reduction (less than 10%) in the T cells proliferation (both CD4^+^ and CD8^+^) compared to the normal saline control group. However, we believe that this effect is negligible and could be due to a background effect/analytical error. Indeed, it is not comparable to the ~50–70% inhibition of T cells proliferation observed with Prograf and TAC-loaded PLGA NPs.

To assess the effect of multiple dosing of TAC (1 mg/kg/day for 30 days), either as Prograf or in the PLGA NPs formulation, on kidney function, serum levels of creatinine, urea, uric acid, and potassium were determined in mice. Empty PLGA and normal saline served as control groups. *In vivo* administration of Prograf to mice showed signs of nephrotoxicity including increased serum creatinine, urea, uric acid, and potassium. These parameters were found to be significantly higher in comparison to the other groups i.e. normal saline, empty PLGA NPs and TAC-loaded PLGA NPs in mice (ANOVA, *p* < 0.05). Although TAC-loaded PLGA NPs also induced the levels of those parameters when compared with normal saline and PLGA NP group, the levels were significantly lower than those obtained with Prograf (Fig. 4). Furthermore, the change in the levels of kidney function parameters in male mice were nearly identical to those obtained in their female counterparts. In order to confirm these findings on a different species, the same experiment was repeated using male and female Wistar rats. The biochemistry and nephrotoxicity profiles were similar to those obtained in mice (Fig. 5). It is worth to mention that the dose administered to rats, although identical in terms of mg/kg, was nearly double the equivalent dose to mice on mg/m^2^ basis^49^.

Histopathological studies revealed a high glomerular congestion with interstitial infiltration and oedema in the kidneys of mice treated with Prograf (Fig. 3C). On the other hand, PLGA NPs were able to significantly reduce the toxicity of tacrolimus, where it only had minimal effects on the kidneys of mice compared to those obtained with control groups i.e. normal saline and empty PLGA NPs (Fig. 3A,B,D). This better nephrotoxicity profile observed for tacrolimus-loaded PLGA NPs over Prograf may suggest that these NPs helped reduce the accumulation of the drug in kidneys. Histopathological findings are in concomitance with serum biochemistry observations.

This work provides another evidence that encapsulation of drugs inside a nano delivery system can help reduce organ toxicity. This has been shown with several delivery systems including nanoparticles, liposomes, polymeric micelles, and others. For instance, Aliabadi *et al*. have shown that encapsulating cyclosporine A inside poly(ethylene oxide)-*block*-poly(ε-caprolactone) significantly reduced the kidney uptake of the drug as well as the nephrotoxicity in mice^50^ without compromising its efficacy^51^. Another example is the capability of the PEGylated liposomes to significantly reduce cardiotoxicity and myelosuppression of the encapsulated doxorubicin (Caelyx/Doxil) while maintaining efficacy in cancer patients^52^.

Taken all together, our developed PLGA NPs were capable of reducing the nephrotoxic effects of TAC in rodent models. This favourable effect occurred without compromising the immunosuppressive effects of the drug as compared to Prograf. Biodegradable nature of the PLGA and its minimal toxicity makes it preferable copolymer for making nano drug delivery systems. Reduction in toxicities and improved efficacy is desired for any drug; however, this will be of significant importance in cases like allogenic organ transplants. In addition to debilitating health conditions of the patients, long-term immunosuppressive therapy itself increases chances of cancer and infections^53,54^.

## Conclusions

The results suggested that the PLGA NPs formulation provides an attractive alternative to the currently available TAC formulation. The developed formulation has the potential to reduce the associated nephrotoxicity while maintaining the immunosuppressive activity of TAC. The results also provide further evidence of the effectiveness of appropriately designed drug delivery systems to enhance the therapeutic index and clinical benefit of existing drugs.

## Materials and methods

### Chemicals and reagents

Tacrolimus was extracted from expired Prograf 5 mg capsules (Batch # 7241), as previously described^55^. Ploy (D, L-lactide-*co*-glycolide; lactide: glycolide (50:50) (PLGA)) with Mw 30,000–60,000 and Poloxamer-188 were purchased from Sigma Aldrich Co. (St. Louis MO, USA). Chloroform and dichloromethane (DCM) were purchased from MERK (Darmstadt, F.R. Germany) and PANREAC QUIMICA SA, (Barcelona, Spain), respectively. Polyvinyl alcohol (PVA, Mw 17,200) and acetone were purchased from AVON- CHEM Ltd. (Wellington House, Waterloo St. West, Macclesfield, Cheshire, UK). The HPLC grade methanol was obtained from BDH Ltd. (Poole, England). Phosphate buffer saline was obtained from GIBCO, Life Technologies Ltd. (Paisley, Scotland). Acetonitrile (HiPerSolv Chromanorm, HPLC-grade) were purchased from BDH, PROLABO1, LEUVEN, EC. Milli-Q water purifier (Millipore, France) was used for purified water and all other used chemicals were analytical grade and solvents were HPLC grade. Diagnostic kits for creatinine, blood urea, uric acid and potassium (Giesse Diagnostics, Rome, Italy).

### PLGA NPs preparation and TAC encapsulation with PLGA NPs

Emulsification-diffusion method was utilized to formulate PLGA-NPs^46^. Briefly, PLGA (100 mg) and TAC (15 mg) were dissolved in 2.5 mL DCM. This mixture (organic phase) was added to 7.5 mL aqueous phase (PVA, 1% w/v and Poloxamer-188, 1% w/v in Milli-Q water). Thereafter, the mixture was emulsified using a homogenizer at a speed of 21,500 rpm for 10 min. The volume of this primary emulsion was increased four times with the addition of aqueous phase with magnetic stirring (1000 rpm). This allowed the organic phase to leave the droplets. Three-hour stirring allowed removal of the excess organic solvent through evaporation leaving drug-loaded PLGA NPs suspension. The obtained suspension of the NPs was then purified by washing with Milli-Q water and isolated by ultracentrifugation (30,000 rpm for 30 min). The process of washing and ultracentrifugation were repeated three times to get the purified NPs. NPs were lyophilized and stored for further studies. Same procedure was followed to prepare empty PLGA NPs (without TAC).

### PLGA NPs characterization

#### Particle size, polydispersity and zeta-potential

The suspension of the purified NPs was diluted with Milli Q water (in 1:5, v/v ratio of suspension of NPs and water) and then were subjected for the measurement of mean particle size, size distribution and polydispersity index by dynamic light scattering (DLS) method using Zetasizer Nano-Series (Nano- ZS90, Malvern Instruments, England) at 25 °C at 90° scattering angle^56^. The software DTS-Version 4.1 (Malvern, England) was used to determine the zeta-potential of the PLGA-NPs and TAC-PLGA NPs.

#### Drug encapsulation efficiency

To determine the encapsulation and loading of TAC into PLGA NPs, around 10.5 mg of drug-loaded PLGA NPs was dissolved in a mixture of chloroform and acetone (1:1, v/v). The solvents were evaporated and obtained residue was dissolved in methanol with sonication and magnetic stirring. Solution was centrifuged at 13,500 rpm for 15 min and supernatant was collected for TAC quantification by HPLC [Waters1 1500 series controller, USA; UV detector (Waters1 2489, dual absorbance detector, USA); pump (Waters1 1525, Binary pump, USA); automated sampling system (Waters1 2707 plus autosampler, USA)] and the system was controlled and monitored by “Breeze (Waters1)” software. TAC was analysed by injecting 30 µL of the supernatant to a C_18_ column (Macherey-Nagel, 4.6 × 150 mm, 10 µm particle size). The mobile phase was consisted of 75:25 (v/v) of acetonitrile and Milli Q water and the pH of water was adjusted to 3 by orthophosphoric acid. The detection wavelength was set at 215 nm, the flow rate of the mobile phase was 1 mL/min and column temperature was set to 60 °C^36,57^. The drug concentration was calculated by using straight line equation: y = 16.784x+ 13529; R^2^ = 0.9993. The encapsulation (%EE) and drug loading (%DL) was calculated with the following equations [Eqs. (1) and (2)]:

1 $$ \% {\rm{Encapsulation}}\,{\rm{Efficiency}}\,({\rm{EE}})=\left(\frac{{\rm{Amount}}\,{\rm{of}}\,{\rm{drug}}\,{\rm{in}}\,{\rm{NPs}}\,({\rm{mg}})}{{\rm{Initial}}\,{\rm{Drug}}\,{\rm{Amount}}\,({\rm{mg}})}\right)\times 100$$

2 $$ \% {\rm{Drug}}\,{\rm{Loading}}\,({\rm{DL}})=\left(\frac{{\rm{Amount}}\,{\rm{of}}\,{\rm{drug}}\,{\rm{in}}\,{\rm{NPs}}\,({\rm{mg}})}{{\rm{Amount}}\,{\rm{of}}\,{\rm{Nanoparticles}}\,({\rm{mg}})}\right)\times 100$$

#### *In-vitro* drug release

The *in vitro* release of TAC from TAC-loaded NP at pH 7.4 was performed by the Eppendorf method by using shaking water bath. TAC NP solution was diluted with phosphate buffer (pH 7.4) as dissolution medium, so that the final concentration of TAC was 100 µg/mL. Each sample was put in Eppendorf tubes of 1.5 mL capacity, which were placed on floating slab and kept in shaking water bath at 37 °C and shaken at a rate of 100 rpm. At 3, 6, 12, 24, 48, 72, 96, 120, 144, 168, 192, 240, 264, 288 h after starting incubation, tubes were taken out from the water bath in triplicate and centrifuged at 11,000 rpm for 30 minutes. Thereafter, the supernatant was taken and 30 µL of it was injected in to the HPLC system for the determination of TAC concentration^36,57^.

#### Scanning electron microscopy

Morphological features of the PLGA NPs were observed by scanning electron microscopy (Carl Zeiss Evo LS10 Cambridge UK). The samples were coated with gold in an Ion Sputter at 20 mA for 6 min. Observation was done at 10–20 kV accelerating voltage, 8.5 mm working distance and at 2.48 KX magnification power.

### *In vivo* TAC nephrotoxicity evaluation in rodents

All the animal procedures were performed according to the NIH guidelines and were reviewed and approved by Institutional Animal Ethical Committee at the College of Pharmacy, King Saud University. BALB/c mice were obtained from animal house facility of College of Pharmacy, King Saud University, Riyadh and were kept in the polypropylene cages with 12 h dark-light cycle at 25 °C, with free access to food and water. The aim of this study was to investigate the potential of PLGA NPs formulation of TAC in reducing the nephrotoxicity of the drug. Thus, we planned the study with four groups of animals from each species with equal number of males and females (n = 6) to receive either Prograf, empty PLGA NP, TAC-loaded PLGA NPs, or normal saline.

All animals weighed and found between 20–25 g (mice). As per the protocol, each animal was supposed to get a daily subcutaneous dose for 30 days. The daily dose of TAC was 1 mg/kg administered as Prograf or TAC-loaded PLGA NPs.

### Histopathological slides preparation

Kidneys from each mouse were collected at the time of termination of the study and immediately were fixed in 10% buffered formalin. Kidneys were processed overnight for dehydration, and then embedded in wax for section cutting in microtome. Five µm thick slices of the kidneys were cut and stained with Haematoxylin and Eosin (H&E). All the slides were analysed using microscope and changes were compared with control group animals.

### Assessment of renal function

Blood was collected from mice from all the treatment groups via cardiac puncture at the time of termination of the experiment. Serum was collected by centrifugation of whole blood at 1000 × *g* for 15 min. Levels of creatinine, urea, uric acid, and potassium were measured using standard diagnostic kits (Giesse Diagnostics, Rome, Italy). Blood was processed immediately after collection and all the parameters were measured on the same day. The same experiment was repeated in male and female Wistar rats (200–250 g).

### Assessment of *in vivo* immunosuppressive activity of tacrolimus-loaded PLGA NPs

The whole blood was collected from four different treatment groups (Prograf, empty PLGA NP, tacrolimus-loaded PLGA NPs, and normal saline; n = 6 mice/group) was subjected to RBC lysis by the addition of RBC lysis solution before flow cytometric analysis. Animals (male mice) received the subcutaneous dose the similar way mentioned above in nephrotoxicity evaluation section except the duration was 7 days. Normal saline was used as a negative control. To assess *in-vivo* immunosuppressive effects of tacrolimus-loaded PLGA NPs, CD4^+^ and CD8^+^ T cells were analysed in whole blood using flow cytometry (LSRII BD biosciences). Peripheral blood mononuclear cells (PBMCs) were isolated from pooled whole blood samples of 5 mice (1.5 mL/mice) using Ficoll density gradient centrifugation. PBMCs were incubated with pre-warmed PBS containing carboxyfluorescein diacetate succinimidyl ester (CFSE) dye (2 μM) for 8 min at 37 °C. Thereafter, cells were washed with PBS and subsequently stimulated with Concanavalin A (ConA) super antigen (10 µg/mL/10^6^ cells). The cells were then incubated for 4 days at 37 °C. After incubation the cells were stained with fluorochrome conjugated anti CD4 or anti-CD8 antibodies specifically targeting T cells among the PBMCs. Standard surface staining protocol was followed for CD4^+^ and CD8^+^ T cells using anti-mouse CD4–APC (R&D systems) and CD8-PE-CY7 (R&D systems) monoclonal antibodies. Frequency of proliferative T lymphocytes was determined using flow cytometry (LSRII BD biosciences) by acquiring up to 1 × 10^6^ events for each condition. Data were then analysed using DIVA software (BD Biosciences).

### Statistical analysis

Data obtained from different treatment groups were statistically analysed using Student’s t-test. Differences in mean values for groups were statistically analysed using analysis of variance (ANOVA) followed by Dennett’s multiple comparisons test (GraphPad Instat software). The target statistical significance was set at *p* < 0.05 for all comparisons.

## Supplementary information

Supplementary info.

## Data Availability

Correspondence and requests for materials should be addressed A. A.
