# Supplementary material for: Mitigation of Tacrolimus-Associated Nephrotoxicity by PLGA Nanoparticulate Delivery Following Multiple Dosing to Mice while Maintaining its Immunosuppressive Activity
Source: Sci Rep. 2020 Apr 21;10:6675. doi: 10.1038/s41598-020-63767-1 (PMC7174389; doi:10.1038/s41598-020-63767-1)
Supplement: Supplementary file 1 — Supplementary info. [file 41598_2020_63767_MOESM1_ESM.docx]

**Supporting Information (SI)**

**Mitigation of Tacrolimus-Associated Nephrotoxicity by PLGA Nanoparticulate Delivery Following Multiple Dosing to Mice while Maintaining its Immunosuppressive Activity**

Aws Alshamsan^1,2§^*, Ziyad Binkhathlan^1,2,3§^, Mohd Abul Kalam^1,2^, Wajhul Qamar^4,5^, Hala Kfouri^6^, Mohammed Alghonaim^7^, and Afsaneh Lavasanifar^3,8^

^1^ Nanobiotechnology Unit, College of Pharmacy, King Saud University, P.O. Box 2457, Riyadh 11451, Saudi Arabia.

^2^ Department of Pharmaceutics, College of Pharmacy, King Saud University, P.O. Box 2457, Riyadh 11451, Saudi Arabia.

^3^ Faculty of Pharmacy and Pharmaceutical Sciences, University of Alberta, Edmonton, Alberta T6G 2H7, Canada.

^4^ Central Laboratory, College of Pharmacy, King Saud University, Riyadh, Saudi Arabia.

^5^ Department of Pharmacology and Toxicology, College of Pharmacy, King Saud University, Riyadh, Saudi Arabia.

^6^ Department of Pathology, College of Medicine, King Saud University, Riyadh, 11451, Saudi Arabia.

^7^ King Salman Bin Abdulaziz Chair for Kidney Disease, King Saud University, Riyadh, 11451, Saudi Arabia.

^8^ Department of Chemical and Material Engineering, University of Alberta, Edmonton, Alberta T6G 2V4, Canada.

^§^ These authors contributed equally to this work

**٭**Corresponding Author − Aws Alshamsan: [aalshamsan@ksu.edu.sa](mailto:aalshamsan@ksu.edu.sa)

**SI Materials and methods**

**PLGA NPs *in vitro* cell uptake**

Dendritic cells (DCs), maintained in aseptic conditions, were incubated with FITC-labelled PLGA NPs for 30 min. After incubation, cells were washed twice with PBS and were subjected to flow cytometry. The flow cytometry analysis was done using 525 nm filter (FL1) with medium flow rate with a cell count of 10^4^. The shift in the fluorescence peak was monitored for uptake of FITC-labelled PLGA NPs. The experiments were run in triplicate. For fluorescent microscopy, the cells were fixed in chilled methanol (-20 °C) and the cells were spread on slides and air dried. The slides were analysed under fluorescent microscope to detect FITC-labelled PLGA NPs uptake into the dendritic cells.

**Supplementary Data**


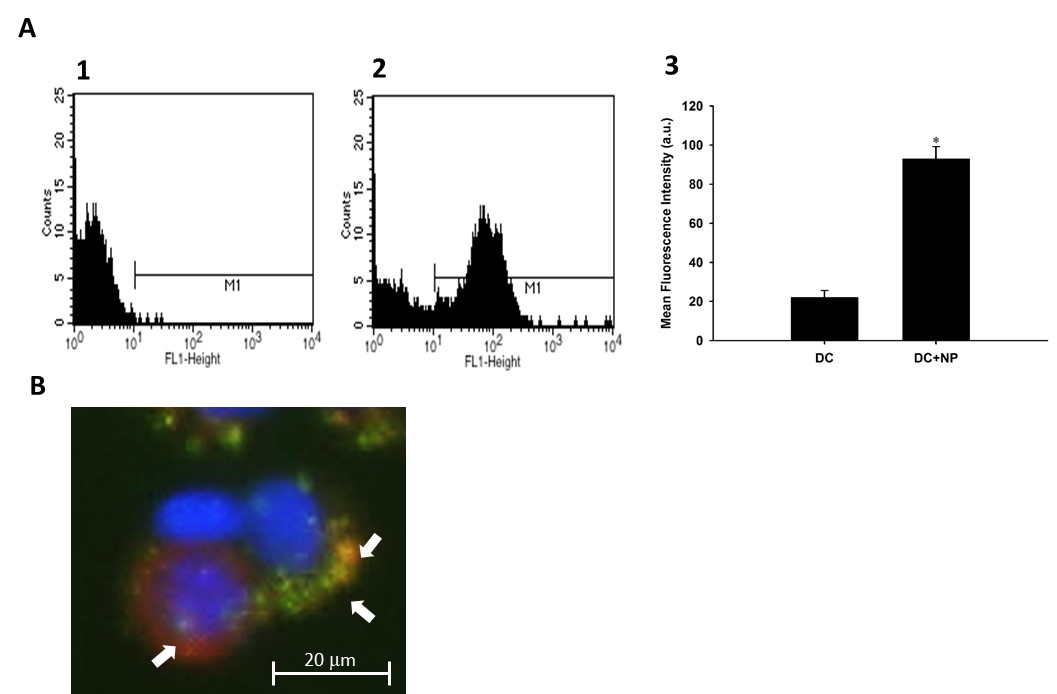


**C**

**B**

**Figure S1.** Cellular uptake of FITC-tagged PLGA NPs was evaluated by flow cytometry: (A) Representative histograms of the untreated control cells (A1) and cells treated with coumarin-6-loaded NPs (A2) can be seen, (B) Mean fluorescence intensity (MFI) of the cells treated with coumarin-6-loaded NPs and coumarin solution is quantified, (C) HT-29 cells incubated with coumarin-6-loaded NP were photographed under fluorescent microscope. Arrow heads indicate several cellular localizations where NPs seemed to be concentrated

Figure S1 (A) shows the flow cytometry data for the *in vitro* cell uptake study in dendritic cells. When FITC-tagged PLGA NPs were added to the dendritic cells, an increase in fluorescence was observed by flow cytometry as compared to untreated cells (Figure S1 A1 and A2). The mean fluorescence intensity associated with DCs incubated with FITC-tagged PLGA NPs was nearly 5-fold higher than that obtained with untreated cells (*p*<0.05, Student’s *t*-test) (Figure S1 B). These findings were confirmed by the fluorescence microscopy of the same cells (Figure S1 C).
